# Low CCL17 expression associates with unfavorable postoperative prognosis of patients with clear cell renal cell carcinoma

**DOI:** 10.1186/s12885-017-3106-y

**Published:** 2017-02-08

**Authors:** Ying Xiong, Li Liu, Yu Xia, Jiajun Wang, Wei Xi, Qi Bai, Yang Qu, Jiejie Xu, Jianming Guo

**Affiliations:** 1 Department of Urology, Zhongshan Hospital, Fudan University, Shanghai, 200032 China; 2 Department of Biochemistry and Molecular Biology, School of Basic Medical Sciences, Fudan University, Shanghai, 200032 China

**Keywords:** Chemokine (C–C motif) ligand 17, Clear cell renal cell carcinoma, Prognostic factor, Overall survival, Recurrence-free survival

## Abstract

**Background:**

Chemokine (C–C motif) ligand 17 (CCL17) is a chemokine mainly produced by myeloid dendritic cells. It is a ligand for CC chemokine receptor 4 (CCR4) and CC chemokine receptor 8 (CCR8). The aim of this study was to investigate prognostic values of CCL17 expression in patients with clear cell renal cell carcinoma (ccRCC).

**Methods:**

The study included 286 patients with ccRCC. CCL17 expression was analyzed by immunohistochemistry on tissue microarrays. Prognostic values of CCL17 expression and patients’ clinical outcomes were evaluated.

**Results:**

Kaplan-Meier method showed that low CCL17 expression was associated with worse patient overall survival (OS) and recurrence-free survival (RFS) (OS*, P* = 0.002; RFS, *P* = 0.007). Low CCL17 expression was an adverse independent risk factor for OS and RFS in multivariate analyses (OS, *P* = 0.006, *P* = 0.011 for bootstrap; RFS, *P* = 0.002, *P* = 0.025 for bootstrap). We constructed two nomograms incorporating parameters derived from multivariate analyses to predict patients’ OS and RFS (OS, c-index 0.799; RFS, c-index 0.787) and they performed better than existed integrated models.

**Conclusion:**

Low CCL17 expression is a potential independent adverse prognostic biomarker for recurrence and survival of patients with ccRCC after nephrectomy. Established nomograms based on this information could help predict ccRCC patients’ OS and RFS.

**Electronic supplementary material:**

The online version of this article (doi:10.1186/s12885-017-3106-y) contains supplementary material, which is available to authorized users.

## Background

Renal cell carcinoma (RCC) is the most common malignant cancer in the adult kidney and accounts for 2 to 3% of all malignancies in adults [1]. Clear-cell RCC (ccRCC) is the major histological subtype according to the WHO classification, which accounts for 80–90% of all RCC patients [2]. Around one third of the patients who underwent curative surgeries would develop recurrences or metastases afterwards [3]. Several prognostic factors and integrated staging systems have been developed for RCC patients such as TNM stage, Fuhrman grade and several integrated models like University of California Integrated Staging System (UISS) and Mayo Clinic stage, size, grade and necrosis (SSIGN) score [4]. Unfortunately, these models are not accurate enough due to the genetic complexity and heterogeneity of the disease [5]. Improved predictive models of survival for ccRCC are needed.

Numerous evidences indicate that chemokines play pleiotropic roles in tumor cell biology [6]. Chemokine (C–C motif) ligand 17 (CCL17), also known as thymus and activation-regulated chemokine (TARC) [7], is a chemokine produced by myeloid dendritic cells, endothelial cells, bronchial epithelial cells and several tumor cells [8]. It is a ligand for CC chemokine receptor 4 (CCR4) and CC chemokine receptor 8 (CCR8). It is able to recruit T cells particularly Th2 cells and activate other antigen-presenting cells [9]. Researchers recently proved that CCL17 was involved in recruiting cytotoxic T cells by binding to CCR4 [10] and activating CD8+ T cells through dendritic cells [11]. These findings indicate that CCL17 is able to enhance antitumor immunity. We wondered whether chemokine CCL17 could act as a promising biomarker candidate for RCC. The role of CCL17 in the development of ccRCC remains unknown so we analyzed the impact of CCL17 expression on patients’ overall survival (OS) and recurrence-free survival (RFS) in a large cohort of ccRCC patients.

## Methods

### Patients and specimens

A total of 286 ccRCC patients who received nephrectomy in Zhongshan Hospital, Fudan University during Jan 2005 and Jun 2007 were enrolled in our study. Clinical Research Ethics Committee of Zhongshan Hospital, Fudan University had approved the study and granted permissions to access the patient records. Written and informed consent was obtained from each individual enrolled in the study. Clinicopathological variables included age, gender, tumor size, TNM stage, Fuhrman grade, Eastern Cooperative Oncology Group performance status (ECOG PS) and necrosis. SSIGN score and UISS score were assessed for each patient. Patients should meet the primary criteria of having pathologically proved ccRCC, having received nephrectomy and having available Formalin Fixed Paraffin Embedded (FFPE) specimen of tumor mass (≥1 cm^3^). Patients who had other former malignant tumors, perioperative mortalities, histories of adjuvant or neo-adjuvant therapies including targeted therapies, mixed type renal cancer or bilateral renal cancer were excluded. Samples with over 80% necrotic or hemorrhagic area were excluded either.

### Data collection

Patients’ OS was defined as the time of nephrectomy to the time of death or last follow up while RFS was calculated from the time of nephrectomy to the time of recurrence. Recurrence was confirmed by imaging, biopsy or physical examination. There were altogether 24 patients excluded from RFS analysis because of missing data of recurrence state or preoperational metastases. Patients were followed up every 3 months during the first 5 years after operation and once a year thereafter. Data was censored until Jan 30, 2015, the last follow up time or the time when patient died. Two pathologists (Yuan J. and Jun H.) reviewed the H&E slides to reconfirm histological subtype, stage, and Fuhrman grade. They confirmed ccRCC histological subtypes according to the 2014 EAU guidelines [2]. Tumor stage was classified according to the 2010 AJCC TNM classification [12]. Fuhrman grade and necrosis were reported according to 2012 ISUP consensus [13]. The SSIGN, UISS and SSIGN localized (Leibovich) score were applied to stratify patients into different risk groups [14–16].

### Immunohistochemistry and evaluation

We constructed tissue microarrays (two cores for one tumor block) with formalin-fixed, paraffin embedded surgical specimens. Immunohistochemical staining was performed on tissue microarrays with protocols described previously [17]. Antibodies against CCL17 (Anti-TARC antibody, ab182793, Abcam, diluted 1/100) and visualization reagent (DakoEnVision Detection System) were used. The specificity of the antibody was confirmed by western blot using RCC cell lines. We used Olympus CDD camera, Nikon eclipse Ti-s microscope (×200magnification and × 400magnification) and NIS-Elements F3.2 software to record the staining results. We took three independent shots and chose the strongest for each tumor core. The intensity of immunohistochemical staining of CCL17 was scored by two urologists unaware of the patients’ clinical features and outcomes using Image-Pro Plus version6.0 software (Media Cybernetics Inc., Bethesda, MD, USA). The pooled IOD mean of the six spots in two tumor cores was regarded as the final staining intensity for each block. We defined IOD score of 8461 as the cutoff value for high and low expression with X-tile software according to the ‘minimum *P*-value method’ on the basis of its relation with OS [18].

### Statistical analyses

Statistical analyses were performed using SPSS Statistics 21.0 (SPSS Inc., Chicago, IL), R software version 3.0.2 with the “rms”, “smoothHR” and “phenoTest” [19] package (R Foundation for Statistical Computing, Vienna, Austria) and Stata (version 12.1; StataCorp LP, TX, USA). Mann-Whitney *U* test, Pearson’s chi-square test, Fisher’s exact test or Cochran-Mantel-Haenszel *χ*2 test was used to compare clinicopathological parameters of the patients. Kaplan–Meier analysis was applied to plot the survival curve. Log-rank test was used to compare patient survival between subgroups. Log-rank *P* values were corrected using the formula proposed by Altman and colleagues [20] since *P* values obtained through “minimum *p* value method” might be overestimated. Numbers at risk were calculated at the beginning of each time period. The Cox proportional hazards regression model was used to perform univariate and multivariate analyses. Besides, 1000 bootstrap resamples were performed for reducing overfitting bias. Two nomograms were constructed to predict the OS and RFS. We calculated concordance index to compare the prognostic or predictive accuracy of different models. Hanley-McNeil test was applied to compare the difference between c-indexes. All statistical tests were 2-sided and *P* < 0.05 was considered statistically significant.

## Results

### Expression of CCL17 and its correlation with clinicopathological characteristics

**Fig. 1:**
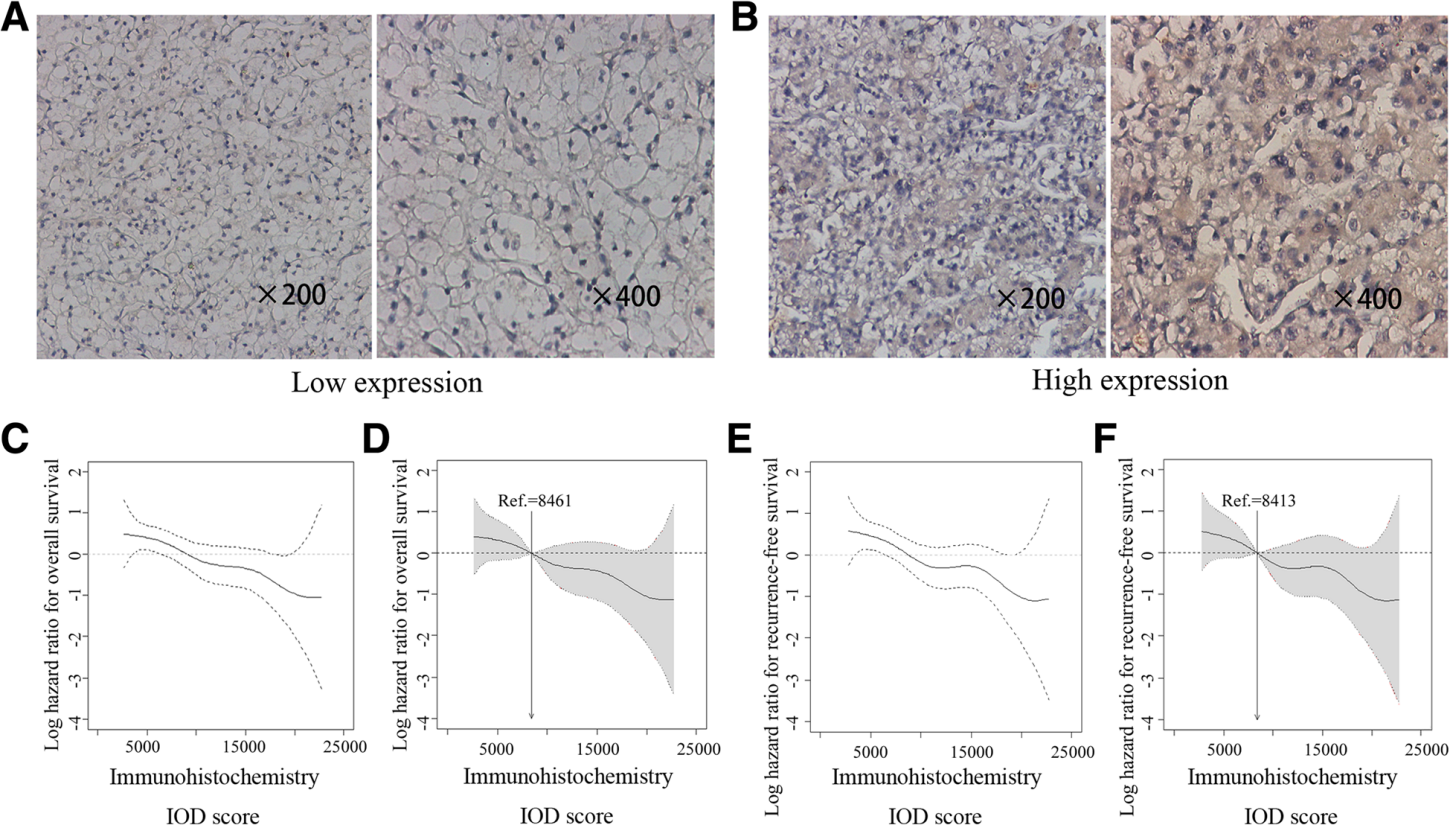
CCL17 expression in clear cell renal cell carcinoma (ccRCC) tissues and Smooth estimates of HR (+1 IOD). Representative CCL17 immunohistochemical (IHC) images of ccRCC tumor tissues with low CCL17 expression (**a**) and high CCL17 expression (**b**). Smooth estimates of HR (+1 IOD) showed a higher risk of death or recurrence for patients with lower CCL17 expression (**c**), (**e**). Smooth estimates of HR (using IOD = 8461 as a reference) showed a significant and stable prognostic difference between patients with high/low CCL17 expression (**d**), (**f**). Dashed lines: 95% confidence bands

We first evaluated CCL17 expression by immunohistochemistry staining analysis in 286 ccRCC patients. CCL17 expression was predominantly found on the cytoplasm of tumor cells and the intensity of the staining was variable (Fig. 1a and b). We illustrated the smooth estimated HR of CCL17 expression (+1 IOD score) on patients’ OS (Fig. 1c). According to the cutoff value (8461) derived from IOD scores and “minimum *p* value method”, 143 of 286 patients were assigned to the low CCL17 expression group and others were assigned to the high CCL17 expression group. The smooth HR curve displayed a significant and stable prognostic difference between the high and low CCL17 expression patient groups with the cutoff value as a reference (Fig. 1d). The curve of smooth estimates did not fluctuate much and log hazard ratio kept decreasing with the increase of IOD scores of CCL17 expression, which indicated that a two-level classification was appropriate (Fig. 1c). CCL17 expression was significantly associated with ECOG PS (*P* = 0.003) while its correlation with other clinicopathological characteristics did not meet statistical significance. The associations between CCL17 expression and clinicopathological features were summarized in Table 1. The median follow-up time was 90.87 months (range 2.63-120.47).

**Table 1:** Clinical characteristics of patients according to CCL17 expression

Characteristics	Patients		CCL17 expression		
	n	%	Low	High	*P*-value
All patients	286	100	143	143	
Age, years					0.811^a^
mean ± SD	55.37 ± 13.24		55.69 ± 13.46	55.05 ± 13.06	
Gender					0.368^b^
Female	87	30.4	47	40	
Male	199	69.6	96	103	
Tumor size, cm					0.173^a^
mean ± SD	4.81 ± 2.67		5.04 ± 2.76	4.58 ± 2.56	
Pathological T stage					0.301^c^
pT1	181	63.3	85	96	
pT2	26	9.1	16	10	
pT3	75	26.2	40	35	
pT4	4	1.4	2	2	
Pathological N stage					1.000^b^
pNx	240	83.9	120	120	
pN0	44	15.4	22	22	
pN1	2	0.7	1	1	
Distant metastasis					0.063^b^
No	271	94.8	132	139	
Yes	15	5.2	11	4	
TNM stage					0.122^c^
I	175	61.2	79	96	
II	23	8.0	13	10	
III	69	24.1	35	34	
IV	19	6.6	14	5	
Fuhrman grade					0.456^c^
1	32	11.2	17	15	
2	209	73.1	99	110	
3	41	14.3	25	16	
4	4	1.4	2	2	
Necrosis					0.866^b^
Absent	245	85.7	123	122	
Present	41	14.3	20	21	
ECOG PS					0.003^b^
0	208	72.7	93	115	
≥ 1	78	27.3	50	28	
UISS category					0.277^c^
Low risk	119	41.6	54	65	
Mediate risk	127	44.4	63	64	
High risk	40	14.0	24	16	
SSIGN category					0.143^c^
0–3	218	76.2	105	113	
5–7	62	21.8	33	29	
8+	6	2.1	5	1	

*ECOG PS* Eastern Cooperative Oncology Group performance status

*P*-value < 0.05 was regarded as statistically significant

^a^Mann-Whitney *U* test for continuous variables, ^b^χ^2^ test or Fisher’s exact test, ^c^Cochran-Mantel-Haenszel *χ* ^2^ test

### High CCL17 expression is associated with better prognosis

**Fig. 2:**
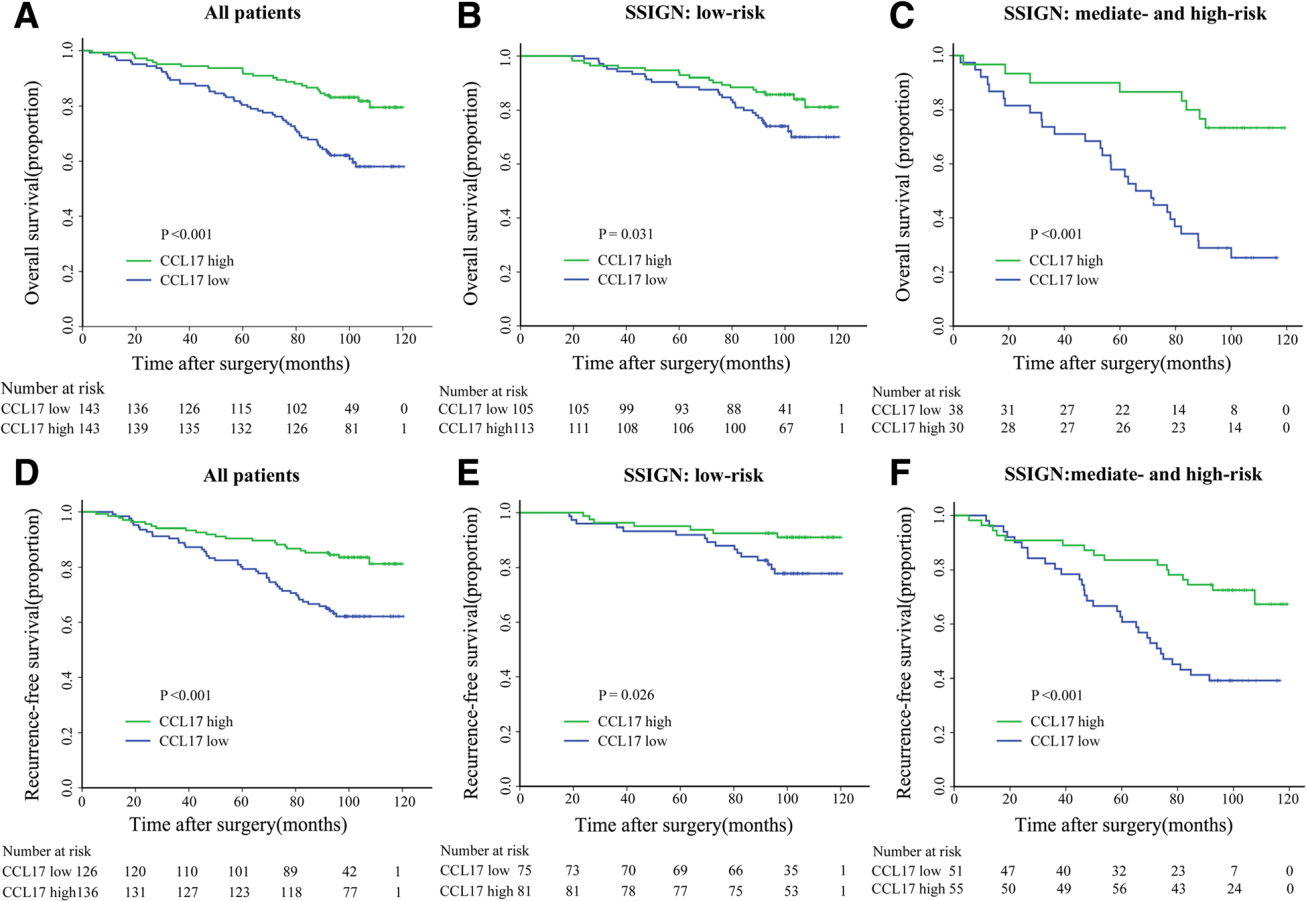
Overall survival (OS) and Recurrence-free survival (RFS) analyses of patients with ccRCC based on CCL17 expression. Kaplan-Meier analysis of OS in All Patients group (*n* = 286) (**a**); and in SSIGN low-risk group (*n* = 218) (**b**); in SSIGN mediate- and high-risk group (*n* = 68) (**c**); Kaplan-Meier analysis of RFS in All Patients group (*n* = 262) (**d**); in SSIGN low-risk group (*n* = 156) (**e**); in SSIGN mediate- and high-risk group (*n* = 106) (**f**). *P* value was calculated by log-rank test

Kaplan-Meier survival analysis was performed to compare OS and RFS according to CCL17 expression. *P* values were corrected [20]. Patients with high CCL17 expression had a significantly better OS (*P* = 0.002) and RFS (*P* = 0.007) than patients with low CCL17 expression (Fig. 2a and d). We then performed univariate and multivariate analyses to further assess whether CCL17 expression was an independent prognostic factor of OS and RFS. Univariate analysis showed that IOD score as a continuous viable was significantly associated with OS and RFS. CCL17 expression as a dichotomous variable was also a risk factor for both OS and RFS in univariate analysis (Additional file 1: Table S1). Furthermore, in multivariate analysis, high CCL17 expression was also a favorable independent risk factor for both OS and RFS (OS, HR, 0.504, 95% CI, 0.309–0.824, *P* = 0.006, *P* = 0.011 for bootstrap; RFS, HR, 0.448, 95% CI, 0.267–0.751, *P* = 0.002, *P* = 0.025 for bootstrap). Pathological T stage, distant metastasis, necrosis, Fuhrman grade and ECOG PS were significantly associated with OS and RFS (Table 2).

**Table 2:** Proportional hazard model for overall survival and recurrence-free survival prediction

Variables	OS (*n* = 286)			RFS (*n* = 262)		
	HR (95%CI)	*P*-value†	*p*-value† (bootstrap‡)	HR (95%CI)	*P*-value†	*p*-value† (bootstrap‡)
Pathological T stage		<0.001			<0.001	
pT1	Reference			Reference		
pT2	2.655 (1.361–5.180)	0.004	0.005	2.586 (1.197–5.587)	0.016	0.011
pT3	2.879 (1.756–4.720)	<0.001	0.001	3.061 (1.795–5.221)	<0.001	0.002
pT4	3.809 (1.054–14.422)	0.027	0.292	8.842 (2.712–28.823)	<0.001	0.009
Distant metastasis						
Yes *vs* No	2.467 (1.283–4.744)	0.007	0.073			
Fuhrman grade		0.005			0.001	
1	Reference			Reference		
2	1.957 (0.701–5.466)	0.200	0.219	1.439 (0.563–3.674)	0.447	0.490
3	4.067 (1.353–12.223)	0.012	0.012	4.024 (1.433–11.301)	0.008	0.019
4	6.786 (1.494–30.828)	0.013	0.002	5.377 (1.258–22.981)	0.023	0.005
Necrosis						
Present *vs* Absent	2.180 (1.216–3.909)	0.009	0.063	2.191 (1.202–3.991)	0.010	0.018
ECOG PS						
0 *vs* ≥1	2.123 (1.333–3.382)	0.002	0.004	2.356 (1.426–3.893)	0.001	0.002
CCL17 expression						
High *vs* Low	0.504 (0.309–0.824)	0.006	0.011	0.448 (0.267–0.751)	0.002	0.025

*ECOG PS* Eastern Cooperative Oncology Group performance status, *HR* hazard ratio, *CI* confidence interval, *OS* overall survival, *RFS* recurrence-free survival

*P*-value < 0.05 was regarded as statistically significant

†Data obtained from the Cox proportional hazards model, ‡Bootstrapping with 1000 resamples were used

### Extension of Prognostic Model with CCL17 expression for ccRCC

As is shown in Fig. 2, the SSIGN/SSIGN (localized) score was applied to classify patients into two risk levels: 0–3 (low), ≥4 (mediate and high) for OS analysis and 0–2 (low), ≥3 (mediate and high) for RFS analysis. High CCL17 expression displayed as a good prognostic factor in intermediated- and high-risk groups in both OS and RFS analyses (OS, *P* = 0.004; RFS, *P* = 0.006). Then, we sought to investigate whether the incorporation of the CCL17 expression into TNM stage, UISS, and SSIGN would improve their predictive accuracy by calculating the C-index (Table 3). CCL17 expression information could add additional power into several existed RCC prognostic models (OS, *P =* 0.003, RFS, *P =* 0.002 for TNM; OS, *P =* 0.006, RFS, *P =* 0.001 for SSIGN; OS, *P* < 0.001, RFS, *P =* 0.001 for UISS) (Table 3).

**Table 3:** Comparison of the predictive accuracy of the prognostic models

Models	Overall survival			Recurrence-free survival		
	C-index (95%CI)	Coefficient (95%CI)	*P*-value	C-index (95%CI)	Coefficient (95%CI)	*P*-value
CCL17	0.615 (0.563–0.667)			0.612 (0.555–0.670)		
TNM	0.706 (0.652–0.760)			0.658 (0.601–0.719)		
TNM + CCL17	0.751 (0.699–0.803)	0.045 (0.016–0.074)	0.003†	0.717 (0.657–0.776)	0.018 (0.022–0.093)	0.002†
SSIGN	0.632 (0.580–0.685)			0.674 (0.617–0.731)		
SSIGN + CCL17	0.679 (0.620–0.738)	0.017 (0.014–0.079)	0.006†	0.720 (0.661–0.778)	0.045 (0.019–0.071)	0.001†
UISS	0.735 (0.688–0.781)			0.710 (0.658–0.762)		
UISS + CCL17	0.771 (0.724–0.818)	0.036 (0.017–0.055)	<0.001†	0.752 (0.697–0.802)	0.047 (0.018–0.065)	0.001†
Nomogram	0.799 (0.754–0.844)			0.787 (0.735–0.840)		
Nomogram *vs* SSIGN		0.167 (0.118–0.215)	<0.001‡		0.109 (0.064–0.155)	<0.001‡
Nomogram *vs* UISS		0.064 (0.030–0.099)	<0.001‡		0.073 (0.031–0.115)	=0.001‡

C-index and 95%CI were calculated from 1000 bootstrap samples to protect from overfitting

*C-index* concordance index, *CI* confidence interval, *SSIGN* Mayo clinic stage, size, grade, and necrosis score, *UISS* UCLA Integrated Staging System

†Compared the c-index with the original model without CCL17 expression data; ‡ Compared the c-index of nomogram with SSIGN/UISS stratification in different patient groups

### Prognostic Nomograms of ccRCC

**Fig. 3:**
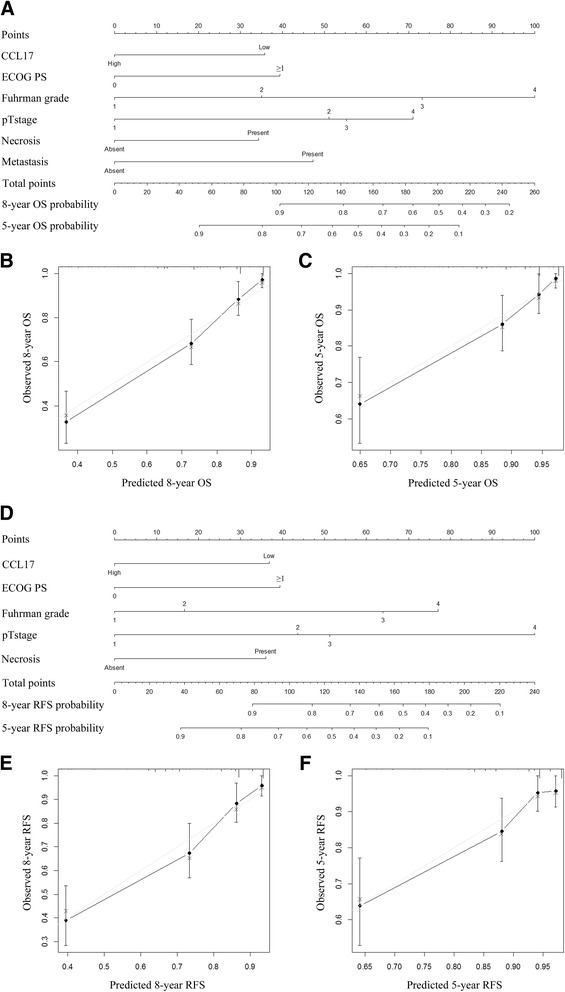
Prognostic nomograms and calibration plots for OS and RFS prediction. Six independent prognostic factors including CCL17 expression, ECOG PS, Fuhrman grade, pathological T stage, necrosis and metastasis were identified and entered into the nomogram (**a**). Calibration curves for predicting 8-year OS of ccRCC patients (**b**). Calibration curves for predicting 5-year OS of ccRCC patients(**c**). Five independent prognostic factors including CCL17 expression, ECOG PS, Fuhrman grade, pathological T stage and necrosis were identified and entered into the nomogram (**d**). Calibration curves for predicting 8-year RFS of ccRCC patients (**e**). Calibration curves for predicting 5-year RFS of ccRCC patients (**f**)

We constructed two nomograms to predict OS and RFS at 5 and 8 years after nephrectomy (Fig. 3a, d). The predictors were based on the validated multivariate analyses (Table 2), including pathological T stage, distant metastasis, Fuhrman grade, necrosis status, ECOG PS and CCL17 expression. The C-index showed a good predictive accuracy for nomograms in both OS and RFS (OS, C-index 0.799; RFS, C-index 0.787). The nomogram to predict OS showed better prognostic capability compared with SSIGN (Nomogram vs SSIGN, *P <* 0.001) and UISS (Nomogram vs UISS, *P* < 0.001). In terms of RFS, the second nomogram also performed better than SSIGN (Nomogram vs SSIGN, *P <* 0.001) and UISS (Nomogram vs UISS *P* = 0.001). Calibration curves for nomogram predicted 5-year and 8-year OS (Fig. 3b, c) and RFS (Fig. 3e, f) were established and the plots displayed good consistency between the predicted and actual observation of patients’ survival.

## Discussion

In this study, we found that CCL17 was predominantly expressed on cytoplasm of tumor cells through immunochemistry, and high CCL17 expression turned out to be positively correlated with a better prognosis. What’s more, CCL17 expression was an independent prognostic factor for OS and RFS of ccRCC patients. The combination of CCL17 expression and current prognostic models like TNM stage, UISS and SSIGN was able to enhance prognostic accuracy. In the end, we generated two nomograms by incorporating CCL17 with other clinicopathological parameters derived from multivariate analysis to predict patients’ OS and RFS. Comparisons by C-indexes showed that the two nomograms performed better than current prognostic models.

As a member of the CC-motif chemokine family, CCL17 is actively secreted by immune cells. CCL17 binds to the G-protein coupled CCR4 and CCR8 and serves for the recruitment and migration of these receptor-expressing cells [21, 22]. CCL17 was considered to attract CCR4+ Treg cells to the tumor. CCL17 created a favorable environment where tumor cells could escape from host immune responses in some type of cancers [23]. Different functions of CCL17 are being discovered these years. CCL17 produced by dendritic cells is able to attract naive cytotoxic T lymphocytes expressing CCR4 and enhance cytotoxicity [10]. It is also a mediator of CD8+ T cell activation through dendritic cells [11]. The prognostic significance of CCL17 varies with the type of malignancy. CCL17 high expression in tumor cells predicts poor survival in patients with hepatocellular carcinoma [24]. Elevated serum level of CCL17 predicts better survival in renal cell carcinoma after peptide vaccination [25] and melanoma carcinoma [26]. Researchers treated 68 subjects with IMA901, a therapeutic vaccine for RCC consisting of multiple tumor associated peptides. Among 300 serum biomarkers, researchers identified that low apolipoprotein A-I and CCL17 predicted worse IMA901 treatment response and overall survival [25]. Our study revealed that low CCL17 expression was also associated with poor patients’ survival without IMA901 treatment.

CCR4 is expressed by CD4+ T cells, CD8+ cytotoxic T lymphocytes, natural killer cells, macrophages and subsets of DCs [27]. Mogamulizumab (KW-0761) is a humanized antiCCR4 mAb approved for treatment of certain types of adult T-cell leukemia and peripheral T-cell lymphoma. A clinical study of mogamulizumab for the treatment of CCR4-negative advanced or recurrent solid cancer is now conducted [28]. CC17 and CCL22 are both ligands of CCR4 but they probably have opposing effects on Treg homeostasis in that CCL22 favors Treg recruitment whereas CCL17 has been reported to convert the Treg phenotype to an inflammatory mediator [29, 30].

However, some limitations remained to be solved. This was a retrospective study in nature and the number of patients enrolled was limited. Though bootstrap internal validation has been used, the issues of over-fitting still existed. Cohort-specific biases including the method of tissue fixation, the staining protocols, the lot of the antibody, preparation and storage of the slides could largely affect our conclusions. Since all patients were from one institution, patient ethnicity/race, clinical practices at the institution and selection biases could also lead to cohort-specific biases thus affected the results. Lack of external validation was the major limitations of our research. We need to validate the results in an independent cohort to support our conclusions in the future. Moreover, the heterogeneity of tumors might also affect the results though we took two tissue cores and took six shots from one tumor block. Further researches were required to investigate the roles of CCL17 in ccRCC tumor cells.

## Conclusion

In conclusion, we have identified that low CCL17 expression was strongly associated with a poor outcome and CCL17 can be used as a novel prognostic factor in predicting patients’ OS and RFS. We also developed nomograms for OS and RFS, which could give a better prediction for patients with ccRCC after surgery.

## Additional file

Additional file 1: Table S1. Univariate analyses of characteristics associated with overall survival and recurrence-free survival. (DOC 58 kb)

## Abbreviations

AJCC: American Joint Committee on Cancer
CCL17: chemokine (C–C motif) ligand 17
CCR4: CC chemokine receptor 4
CCR8: CC chemokine receptor 8
ccRCC: Clear cell renal cell carcinoma
ECOG PS: Eastern Cooperative Oncology Group performance status
OS: Overall survival
RFS: Recurrence-free survival
SSIGN: Mayo Clinic stage, size, grade and necrosis
TARC: Thymus and activation-regulated chemokine
UISS: University of California Integrated Staging System
