# Supplementary material for: Low CCL17 expression associates with unfavorable postoperative prognosis of patients with clear cell renal cell carcinoma
Source: BMC Cancer. 2017 Feb 8;17:117. doi: 10.1186/s12885-017-3106-y (PMC5299767; doi:10.1186/s12885-017-3106-y)
Supplement: Additional file 1: Table S1. — Univariate analyses of characteristics associated with overall survival and recurrence-free survival. (DOC 58 kb) [file 12885_2017_3106_MOESM1_ESM.doc]

**Table S1: Univariate analyses of characteristics associated with overall survival and recurrence-free survival**

| **Variables** | **OS (n=286)** | | |  | **RFS (n=262)** | | |
| --- | --- | --- | --- | --- | --- | --- | --- |
|  | **Hazard Ratio** | **95%CI** | **P-value**† |  | **Hazard Ratio** | **95%CI** | **P-value**† |
| Age, years | 1.029 | 1.012-1.047 | <0.001 |  | 1.025 | 1.007-1.044 | 0.008 |
| Gender |  |  | 0.901 |  |  |  | 0.944 |
| Male *vs* Female | 1.030 | 0.645-1.647 |  |  | 0.982 | 0.53-1.627 |  |
| Tumor size (continuous) | 1.163 | 1.088-1.244 | <0.001 |  | 1.143 | 1.059-1.234 | <0.001 |
| Pathological T stage |  |  | <0.001 |  |  |  | <0.001 |
| pT1 | 1.000 | Reference |  |  | 1.000 | Reference |  |
| pT2 | 3.632 | 1.904-6.929 | <0.001 |  | 3.319 | 1.574-7.001 | 0.002 |
| pT3 | 3.386 | 2.094-5.476 | <0.001 |  | 3.064 | 1.820-5.159 | <0.001 |
| pT4 | 7.774 | 2.368-25.528 | 0.001 |  | 15.126 | 5.217-43.850 | <0.001 |
| N classification |  |  |  |  |  |  |  |
| N1 *vs* N0 | 1.669 | 0.218-12.792 | 0.622 |  |  |  |  |
| Distant metastasis |  |  |  |  |  |  |  |
| Yes *vs* No | 6.146 | 3.326-11.357 | <0.001 |  |  |  |  |
| Fuhrman grade |  |  | <0.001 |  |  |  | <0.001 |
| 1 | 1.000 | Reference |  |  | 1.000 | Reference |  |
| 2 | 2.177 | 0.787-6.021 | 0.134 |  | 1.472 | 0.582-3.722 | 0.414 |
| 3 | 6.400 | 2.217-18.479 | 0.001 |  | 5.025 | 1.883-13.409 | 0.001 |
| 4 | 6.099 | 1.364-27.262 | 0.018 |  | 4.914 | 1.173-20.580 | 0.029 |
| Necrosis |  |  |  |  |  |  |  |
| Present *vs* Absent | 2.617 | 1.605-4.266 | <0.001 |  | 3.051 | 1.814-5.132 | <0.001 |
| ECOG PS |  |  |  |  |  |  |  |
| 0 *vs* ≥1 | 3.173 | 2.06-4.886 | <0.001 |  | 3.134 | 1.956-5.023 | <0.001 |
| IOD score of CCL17 expression | 1.000 | 1.000-1.000 | =0.001 |  | 1.000 | 1.000 | 0.002 |
| CCL17 expression |  |  |  |  |  |  |  |
| High *vs* Low | 0.387 | 0.243-0.617 | <0.001 |  | 0.397 | 0.241-0.655 | <0.001 |

ECOG PS= Eastern Cooperative Oncology Group performance status; CI=confidence interval; OS= overall survival; RFS= recurrence-free survival; †Data obtained from the Cox proportional hazards model, P-value <0.05 was regarded as statistically significant
